# Newborn screening for pompe disease? a qualitative study exploring professional views

**DOI:** 10.1186/1471-2431-14-203

**Published:** 2014-08-14

**Authors:** Carla G van El, Tessel Rigter, Arnold JJ Reuser, Ans T van der Ploeg, Stephanie S Weinreich, Martina C Cornel

**Affiliations:** 1 Department of Clinical Genetics/EMGO Institute for Health and Care Research, Section Community Genetics, VU University Medical Center, Van der Boechorststraat 7, 1081BT Amsterdam, The Netherlands; 2 Center for Lysosomal and Metabolic Diseases, Erasmus MC University Medical Center, Wytemaweg 80, 3015CN Rotterdam, The Netherlands; 3 Department of Clinical Genetics, Erasmus MC University Medical Center, Dr. Molewaterplein 50, 3015 GE Rotterdam, The Netherlands; 4 Department of Pediatrics, Division of Metabolic Diseases and Genetics, Erasmus MC University Medical Center, Dr. Molewaterplein 60, 3915GJ Rotterdam, The Netherlands; 5 Centre for Medical Systems Biology, Leiden University, Postbox 9600, 2300 RC Leiden, The Netherlands; 6 CSG Centre for Society and the Life Sciences, Toernooiveld 216, 6525 EC Nijmegen, The Netherlands

## Abstract

**Background:**

Developments in enzyme replacement therapy have kindled discussions on adding Pompe disease, characterized by progressive muscle weakness and wasting, to neonatal screening. Pompe disease does not fit traditional screening criteria as it is a broad-spectrum phenotype disorder that may occur in lethal form in early infancy or manifest in less severe forms from infancy to late adulthood. Current screening tests cannot differentiate between these forms. Normally, expanding screening is discussed among experts in advisory bodies. While advisory reports usually mention the procedures and outcome of deliberations, little is known of the importance attached to different arguments and the actual weighing processes involved. In this research we aim to explore the views of a wide range of relevant professionals to gain more insight into the process of weighing pros and cons of neonatal screening for Pompe disease, as an example of the dilemmas involved in screening for broad-spectrum phenotype disorders.

**Methods:**

We conducted 24 semi-structured interviews with medical, lab, insurance and screening professionals, and executive staff of patient organisations. They were asked about their first reaction to neonatal screening for Pompe disease, after which benefits and harms and requirements for screening were explored in more detail.

**Results:**

Advantages included health gain by timely intervention, avoiding a diagnostic quest, having a reproductive choice and gaining more knowledge about the natural course and treatment. Being prepared was mentioned as an advantage for the later manifesting cases. Disadvantages included treatment costs and uncertainties about its effect, the timing of treatment in later manifesting cases, the psychological burden for the patient-in-waiting and the family. Also the downsides of having prior knowledge as well as having to consider a reproductive option were mentioned as disadvantages.

**Conclusion:**

When weighing pros and cons, interviewees attach different importance to different arguments, based on personal and professional views. Professionals expect benefits from neonatal screening for Pompe disease, especially for early-onset cases. Some interviewees valued screening in later manifesting cases as well, while stressing the need for adequate support of pre-symptomatic patients and their families. Others considered the psychological burden and uncertainties regarding treatment as reasons not to screen.

## Background

In neonatal screening early detection of serious childhood disorders allows for interventions that can prevent or postpone irreversible health damage in the infant. Over the years, in many countries the number of disorders screened for has expanded [1]. National arrangements for discussing and preparing expansions of screening programs vary and have changed over time. In general, expanding screening is discussed among a restricted number of medical and health-policy experts in advisory bodies. Ideally, the WHO Wilson and Jungner criteria or adaptations of these criteria are observed when weighing pros and cons of a potential new screening [2]. These criteria help to assess whether the expected benefits surpass the potential harm of screening. For instance the availability of a treatment to achieve health gain from screening or intervention is essential when deciding on the potential benefit of screening. While advisory reports usually mention the procedures and outcome of deliberations, little is known of the importance of different arguments and the actual weighing processes involved.

In this research we aim to explore the views of a wide range of relevant professionals to gain more insight into the process of weighing pros and cons of neonatal screening. We focus on the case of glycogen storage disease type II, or Pompe disease, a severe autosomal recessive lysosomal storage disorder [MIM ID #232300], involving progressive muscle weakness and respiratory failure.

Developments in treatment via enzyme replacement therapy (ERT) and tests have kindled discussions on adding Pompe disease to neonatal screening. Some jurisdictions have introduced pilot screening programmes for Pompe disease. In Taiwan, screening started in 2005 [3], in Austria a pilot has been conducted [4] and in the USA several states have started screening [5]. Based on an evidence report [6] in May 2013 the US Secretary’s Discretionary Advisory Committee for Heritable Disorders in Newborns and Children (DACHDNC) advised adding Pompe Disease to the recommended uniform screening panel (RUSP) for newborns (recommended to all states) [7], though, at the time of writing, May 2014, the Secretary’s decision is pending.

Pompe disease is particularly interesting as an example of the dilemmas involved in screening for broad-spectrum phenotype disorders. So far, with a few recent exceptions, neonatal screening has been restricted to childhood-onset disorders, where early detection and intervention or treatment can prevent irreversible health damage [8]. Current screening tests for Pompe disease not only detect the classic infantile form - lethal in the first years of life, when untreated – but also less severe cases, for which the age of onset might vary from infancy to adulthood [9]. Since the traditional focus of neonatal screening does not fit well with the potential outcome of this new kind of screening for broad-spectrum disorders, introduction of neonatal screening for Pompe disease would need to be carefully considered from all angles, and screening criteria might need rethinking [10,11].

Recently, the inclusion of views of stakeholders and members of the general public in policy deliberations has been promoted [12,13]. This is expected to increase transparency, accountability and quality of decision making, since it brings in knowledge and views that would otherwise be unheard. Elsewhere we have made a quantitative comparison of patients’ and the general public’s views on expanding neonatal screening for Pompe disease [14]. Here we explore the views of a wide range of relevant professionals. We selected professionals that were either knowledgeable on several aspects of Pompe disease, covering as much as possible the range of expertise involved in the continuity of care for this disease, or were involved in the organisation of screening or health care, including executive staff of patient organisations. Given these diverse experiences and kinds of expertise we expected these professionals to be able to develop an informed opinion on pros and cons of this potentially new type of screening. Because of the need to explore arguments we have chosen semi-structured interviews as a research method.

In the Netherlands, since 2007 the national neonatal screening programme has been expanded from 3 to 19 disorders based on discussions held in 2005 [15]. Pompe disease was considered as a candidate for neonatal screening, but incorporation in the programme was declined because of insufficient evidence of the effect of treatment and uncertainties regarding the availability of treatment at that time. It was stated though, that further developments might merit reconsidering screening for Pompe disease [15]. A study from 2003 reported that in the Netherlands classic infantile cases of Pompe disease are diagnosed at a median age of about 5 months [16] when severe, irreversible muscle weakness has already occurred. Earlier detection would allow for earlier treatment and better health status in infants with this classic infantile form of the disease [17]. However, through screening late manifesting cases would also be detected. This would create a group of pre-symptomatic patients, or patients-in-waiting [18]. In the Netherlands patients with later manifesting forms of Pompe disease are treated with infusions once every two weeks starting when patients show significant signs of muscle weakness or respiratory failure. ERT is very costly, dosage depends on weight. Costs per adult patient vary between roughly 400.000 and 700.000 Euro a year [19]. Currently the costs are covered through basic health insurance. The Ministry of Health has started price negotiations with the drug manufacturer while also promoting more efficacious drug use through various measures [20].

### Objective

Given rapid developments in understanding etiology, test-development, and treatment options for broad-phenotype disorders such as Pompe disease, in this research we explore the views of a wide range of relevant professionals to gain more insight into the process of weighing pros and cons and the importance attached to different arguments in considering neonatal screening for Pompe disease. The aim is to increase transparency and stimulate informed policy-making in expanding neonatal screening, especially of broad-phenotype disorders.

## Methods

### Sample

We selected 24 professionals (see 'List of professional background of interviewees' below) who had experience with various aspects of Pompe disease or had prior knowledge of this disorder (such as paediatricians, neurologists, physiatrists, family doctors), and health care policy officials who were knowledgeable about screening (such as representatives from the Netherlands’ Centre for Population Screening, the Health Care Insurance Board, and a well-baby clinic doctor). Also executive staff members of two patient organisations (respectively dedicated to neuromuscular and metabolic diseases) who have members with Pompe disease were included. We selected this purposeful sample because we expected these professionals to be capable of developing informed opinions regarding the pros and cons and consequences of screening for Pompe disease for patients, their family members, health care and/or wider society. The experts were selected through initial contacts at the national Center for Lysosomal and Metabolic Diseases, at Erasmus MC University Medical Center, that treats all Dutch Pompe patients, and further suggestions via snowballing. Interviewees came from various regions in the Netherlands and those working in academic medical centers were employed by 4 of the total amount of 8 of these centers.

### List of professional background of interviewees

Medical professionals:

Neurologist (2)

Pediatrician (3)

General practitioner (2)

Physiotherapist (2)

Pulmonologist (1)

Clinical geneticist (1)

Physiatrist (2)

Midwife (1)

Well-baby clinic physician (1)

Other experts:

Neonatal screening organisation staff member (2)

National Institute for Public Health and the Environment (RIVM) executive staff member (2)

Patient organisation executive staff member (2)

Health insurance official (1)

Clinical chemist (2)

### Design

Between 2009 and 2011, we conducted 24 semi-structured interviews based on a protocol developed on the basis of our previous study of screening criteria in the Netherlands [21], recent literature on expanding neonatal screening and developments in research and treatment of Pompe disease. The protocol was tested on 2 key experts and made more structured. Prior to the interviews the remaining 22 interviewees received concise written information on Pompe disease, screening and treatment (see Additional file 1). They were informed that currently classic-infantile cases are diagnosed at a median age of about 5 months. In addition, it was verbally explained that in the Netherlands currently 180.000 infants have a heel prick each year. In case of neonatal screening for Pompe disease, 1-2 classic infantile cases, and 3–4 cases with less severe forms were to be expected each year, and an additional 80 false positive cases. Information was given on current procedures in the Netherlands to start treatment by biweekly infusions in later onset cases when patients show significant signs of muscle weakness or decreased pulmonary function. We did not provide a list of screening criteria, such as an overview of the Wilson and Jungner criteria, that are often used to weigh pros and cons. Rather we focused our questions on the potential benefits and harms of screening in the specific case of Pompe disease, thereby exploring the different consequences of screening for classic infantile cases and the later manifesting forms. After discussing their experiences with Pompe patients or screening in general, interviewees were asked about their first reaction to the idea of neonatal screening for Pompe disease, after which benefits and harms for both classic infantile as well as less severe later onset cases were discussed, and pros and cons were weighed. When opportune, requirements for screening were explored.

### Data collection and analysis

The first interviews and some later interviews were conducted by two researchers jointly (TR and CE), the other interviews by one researcher (TR). The interviews were tape-recorded and transcribed literally by a third party. On the basis of the interview protocol a code list was developed (TR and CE): themes were identified and grouped under headings related to ‘introduction’, ‘experiences’, ‘opinions’, ‘advantages’, ‘disadvantages’, ‘prerequisites of screening’, and ‘continuity of care’. The first interviews were coded separately by two researchers (TR and CE), the codes were compared and discussed in case of differences until agreement was reached. Later interviews were coded by one researcher (either TR or CE), and unclear sections were discussed with the other researcher until agreement was reached. During this process some codes were refined or expanded.

### Ethical approval

This study forms part of a larger project which was approved by the institutional review boards of the Erasmus MC University Medical Center (MEC2007-103, addendum 3) and the VU University Medical Center (letter 2010/104). However, interviews with professionals on their opinion regarding health care are considered exempt from strict requirements for ethical approval.

## Results

Relevant and recurrent themes are discussed below. They fall under the following headings: first reaction, benefits and harms for classic infantile and later manifesting cases, weighing pros and cons and prerequisites for screening. We have selected citations to illustrate the views and arguments used within the themes. The number of the interview is indicated between brackets.

### First reaction

After the introduction, when asked about their first reaction whether Pompe disease should be screened for neonatally several interviewees expressed their support, while some are unsure, and others stated their reservations or objections about screening.

Yes, I am strongly in favour of that.

(#5 Medical professional)

I have doubts.

(#8 Other expert)

Then I would say, no.

(#12 Medical professional)

### Benefits and harms of screening in case of classic infantile cases

When asked for arguments for their opinion in terms of benefits of screening for classic infantile cases, respondents often mentioned shortening the diagnostic delay and subsequent health gain.

The real gain, I think, are those children, those babies (…) whom you discover right away, and whom you can start treating right away.

(#4 Medical professional)

In some interviews having reproductive options for the next pregnancy was discussed, and sometimes mentioned as a benefit.

I think it would be a criminal act to burden one family with 2 children with a severe muscular disorder if we have the technology to prevent this. (…) I think this is a big advantage, because then you can prevent it happening again.

(#5 Medical professional)

However, as some other interviewees remarked, since the median age of onset in classic infantile cases has been reported to be 5 months of age, already in the current situation without neonatal screening parents of classic infantile Pompe disease patients would have been informed early enough to allow for reproductive options for future pregnancies.

As for the drawbacks, respondents mentioned uncertainty about the evidence and long-term effects of early treatment. It was known at the time that not all patients responded equally well to treatment, and that some patients experienced allergic reactions. For some this would be a reason not to screen, while others considered this as something that should be kept in mind while screening.

…what is happening is really very spectacular (…), but I myself am not yet convinced of the end result, that the end result will be good enough to warrant screening.

(#7 Other expert)

You cannot predict per person, much more research needs to be done on that, but you see there are children who are doing very well, children who are doing well and there are children who are doing well and then have an enormous dip, get pneumonia, start mechanical ventilation and become rather physically handicapped at age three or four. (…) For the adults we see that the majority still responds well. There are differences there too, some can stand and walk again and do not need (…) a cane any more, others can walk, and another one still has problems. And rarely an individual has an allergic reaction.

(#20 Other expert)

A respondent remarked that new, treatment-independent symptoms were seen that were not known before because previously, patients died at an early age. It was stated that it is a disadvantage that in fact this part of the ‘natural history’ of the disorder is not completely known yet.

…patients that are treated are developing symptoms that we did not know existed in relation to Pompe disease and they will not be treatment-dependent, they belong to the whole spectrum… but … they died and so you did not see these problems, hearing problems, for instance.

(#6 Medical professional)

Also costs were mentioned as a drawback, and were often seen as something to be resolved by parties such as health care insurers or something to be discussed by society at large. Some interviewees expected prices of treatment to drop in the future.

### Benefits and harms of screening in later manifesting, less severe, cases

Some respondents expected clear benefit from early detection through screening for the less severe cases. The ability to monitor and avoid a diagnostic odyssey should health problems develop was seen as an important advantage.

*Another advantage is that you could start treatment in late onset patients in a timely manner.*(*…) They always become ill. And when those patients fall ill, they often have been ailing for 5 to 10 years. And I think it is unethical – if you can diagnose - to think I will wait until health complaints develop.*

(#5 Medical professional)

For parents knowing what might be the matter with their child in case of health problems and knowing what to expect was mentioned as an advantage.

And therefore that is a big advantage of such a screening, that parents have some more clarity, on what is the matter with their child. And when I argue this way, we just took myself as an example, I would not want to know, but I would want to know in case of my child.

(#22 Medical professional)

It was however also mentioned as a drawback that in case of Pompe disease a precise prediction of the disease development cannot be given.

One interviewee saw the possibility to start physiotherapy by means of prevention as an advantage, though it was clear that more research was needed to find out what exercise programmes would work best.

…if I speak from my professional field I think you can start with exercises for specific muscle groups, so you can …prevent potential muscle damage.

(#11 Medical professional)

Others were more sceptical about the effects of physiotherapy.

Another advantage did not relate to the individual patient but to research. By monitoring ‘patients-in-waiting’ after a positive screening more knowledge can be obtained about the development of the disorder but also about administering ERT treatment.

*I also hope that in the future it will become more clear… what … schedule for administering (treatment CE)… has the most effect. Probably much can be gained there as well*.

(#14 Medical professional)

Being prepared was also seen as an advantage. People might be able to anticipate future decisions regarding education, sports or employment.

Choice of profession (is) of course also of paramount importance. Because if you know this (the disease CE) is coming, then you can say: I should certainly not become a gardener.

(#20 Other expert)

However, having prior knowledge was sometimes mentioned as a disadvantage as well.

I think for very many people, the majority, it is an advantage that you can organise your life in relation to what will happen in the future. But of course that has disadvantages. Indeed if you look at education, employment and such, that you do not know when something is going to happen and that you already do make choices, maybe make the wrong choices. Or do not make a choice you prefer more. (…)

(#22 Medical professional)

A disadvantage could be (…) I do not enter into this relationship or I would like to have children but I won’t because I know I will deteriorate in the future.

(#22 Medical professional)

Furthermore, many interviewees stated that the fact the screening would not differentiate between classic infantile cases and later manifesting, less severe forms of Pompe disease was a serious drawback because of the psychological burden this would bring to the patient and his or her family.

*And there is nothing you can…? No, then I would not want to screen. Then I would not want to know. It is an enormous burden (…) You have your child, you are happy… and then you get the verdict (…) But we cannot do something about it yet, only when your child becomes hypotonic, then we can start acting. And then I wonder whether that is of any use*.

(#23 Medical professional)

The psychological burden included informing the child of his or her future health status.

…*do you have to put this burden on the parents…and the question whether they should tell their children and in what phase they should tell them?*

(*#14 Medical professional)*

For parents of patients with a potentially late disease manifestation having reproductive options in future pregnancies was also seen by some as a drawback. Especially since the age of onset and severity of symptoms are largely unpredictable in such cases. However, some interviewees saw having a reproductive choice as an advantage in these cases as well.

From a policy perspective, concerns were raised about the robustness of the neonatal screening programme in case screening for Pompe disease would be added.

…you should see how it would relate to the current package. In some way it has to be uniform…to enable clear information and to make sure participants can make a good choice…. But that would imply they need some knowledge: what disorders do I screen, what does it mean, what are potential consequences? And, of course, with Pompe disease that is a difficult story to tell, so that is something you should really look into, if that story can be told well.

(#15 Other expert)

Other questions and concerns were raised in relation to pre-symptomatic patients and difficulties with obtaining health care insurance, life insurance and employment.

### Weighing pros and cons

After asking for a first reaction and exploring benefits and disadvantages of screening, respondents were invited to weigh pros and cons. Several interviewees thought the benefit for classic infantile cases was more important than the burden for later onset cases

I am afraid that if I must weigh…that the early infantile (cases) have so much benefit, the gain is so big that if we reach them in time, that unfortunately it is worth the burden.

(#3 Medical professional)

However, some respondents voiced a clear vote against screening when weighing pros and cons.

…you create many more problems than you solve if you do it without being able to differentiate between serious forms that need immediate treatment (and) forms that will develop complaints only in 40 years…..I think you will encounter opposition on many fronts, and definitely morally-societally so to speak, if you have (…) a lot of people whose parents have been told: yes your child has Pompe disease, but we do not know yet if health problems will develop in 10, 15 or 40 years…..

(#17 Other expert)

While a few respondents were critical towards neonatal screening for Pompe disease throughout the interview and opted against screening when weighing pros and cons, some others were positive throughout the interview towards neonatal screening and opted in favour of screening. However, a few interviewees initially were positive and changed their opinion into a negative stance towards screening during the interview, after having considered the advantages and disadvantages in more detail. This change of attitude seems mostly related to the fact that the screening test would not be able to differentiate between classic infantile cases and less severe cases of Pompe disease manifesting at unknown age. The interviewees felt that the burden of living in uncertainty about the age of onset of a life threatening disease would be too high.

### Prerequisites

Supporters of screening as well as those who had doubts about or objections to screening formulated prerequisites for screening that were often directly related to overcoming the perceived drawbacks. More evidence on the effect of treatment was desired, though screening was sometimes mentioned as a means to obtain more evidence, for instance via a pilot. A test that would be able to differentiate between classic infantile Pompe disease and less severe forms was found desirable and for some would be required before screening could be considered. In the mean time it was suggested that research might focus on alternatives for screening that would allow for an earlier diagnosis and rapid transfer to specialized care after symptoms would be detected by well-baby clinics or family doctors.

Other requirements that were mentioned were that the test characteristics needed to be optimised in order to reduce the expected number of 80 false positives. The amount of time needed between a positive test result and a second test to confirm or exclude Pompe disease should be as short as possible, according to the interviewees. Parents who would receive a positive result were said to need adequate support, not only immediately after the diagnosis, but also in later years to help them cope with the implications. With regard to diagnosed patients-in-waiting some interviewees argued that psychological support should also be available.

Other considerations concerned potential discrimination against pre-symptomatic Pompe patients in health and life insurance, issues that need to be addressed before screening could be implemented.

Given the sensitive character of the screening both in terms of ethical considerations and cost, some mentioned a wider societal debate would be relevant. It was also mentioned that industry was interested in neonatal screening, and therefore an independent process of policy making should be safeguarded.

The practicalities of the screening process were also discussed. It was mentioned that the midwives who give the information in the last trimester of pregnancy on the heel prick programme, as well as the screeners who in many cases actually perform the heel prick need to be able to explain the complicated outcomes of the screening. The potential outcome of a ‘pre-symptomatic patient’ is perceived as difficult to explain by professionals and difficult to understand for parents, as it falls outside the scope of the current screening programme. This issue merits special attention before implementation, and better education of screening professionals could therefore also be considered as a prerequisite for screening.

## Discussion

In the interviews we conducted with professionals from various backgrounds in health care and neonatal screening programs as well as patient organisation executive staff members we were able to explore and discuss a range of advantages and disadvantages of and prerequisites for neonatal screening for Pompe disease. Advantages for both classic infantile and later manifesting cases included health gain, avoiding a diagnostic quest, having a reproductive choice in future pregnancies, and the ability to gain more knowledge about the disease and treatment. Being prepared was mentioned as an advantage for the later manifesting cases. Disadvantages for both ends of the spectrum included costs and uncertainties about the effect of treatment. In later manifesting cases most notably the timing of treatment and the psychological burden for the patient-in-waiting and the family were seen as major drawbacks. Also the downsides of having a reproductive option were mentioned. Ideally a test should yield only limited false positive and false negative cases and be able to differentiate between the different forms of Pompe disease. However, for some interviewees the lack of discriminative power was not a reason not to screen. Other requirements included proper information and support for parents, education for health care professionals and screeners.

We also gained insight into the process of weighing pros and cons. Individuals draw different conclusions during this process. Some would opt for screening since for them the benefit for classic infantile cases outweighs the potential burden for patients with a late disease manifestation. Others would decline screening because they are of the opposite opinion, or for them there would be too many uncertainties as to the evidence and onset of treatment.

### Strength and limitations

A strength of this study is the fact that we were able to interview a broad range of professionals and executive staff of patient organizations knowledgeable on screening and/or health care for Pompe disease patients. A weakness is that interviewees differed in the amount of detailed knowledge about the latest published and unpublished evidence on efficacy and failures of enzyme therapy. Though most of them were knowledgeable about Pompe disease and its treatment, we cannot be sure whether that has affected the outcome. It should be noted that more evidence on the effect of treatment via enzyme replacement therapy was published after most of the interviews were conducted [22], and the lack of evidence at that time may have led to a cautious (or optimistic) stance. However, uncertainties regarding cases in which treatment is not effective remain and long-term follow up would be necessary to answer questions concerning the right time to start treatment in later manifesting, slowly progressive cases and the effects of early treatment in classic infantile cases.

As we purposely did not provide a list of screening criteria in advance, not all criteria received similar attention. For instance, while the availability of an effective treatment was discussed in detail, the acceptability of treatment hardly surfaced. We do not think this is because of an unfamiliarity with the burden of the bi-weekly infusions. Further research is needed to better understand the relevance of this criterion on weighing pros and cons.

In this qualitative research we did not attempt to relate the opinion on whether to screen or not to a particular professional background. This possibility would, however, be interesting to study further, given the fact that some professional groups traditionally have been more involved with neonatal screening policy, and their arguments and opinions would perhaps be more influential than those of other professional groups. In our sample, interestingly, opinions sometimes varied within one specific subset of interviewees. For instance, the executive staff member of one patient organisation was in favour of screening, whereas the executive staff member of another patient organisation had serious objections to screening. This might support earlier findings of diverging views on neonatal screening among (parents) of Pompe disease patients [14]. This study does not reflect the weighing processes that would occur in an actual advisory committee. Then all members would be presented with the same evidence, and furthermore they would be able to discuss and influence each other’s opinions. However, in the case of Pompe disease not only evidence, but also ideas about the ethical and social ramifications of screening play an important role, as we will argue below.

### Screening in children’s best interest

Traditionally the diseases incorporated in the neonatal screening program are disorders for which early detection can lead to prompt intervention or treatment in order to prevent irreversible health damage to the infant. In the case of screening for Pompe disease, however, the focus on childhood disorders would be loosened as Pompe disease is a broad-phenotype disorder and symptoms may present at any age, including in adulthood.

In the case of neonatal screening for Pompe disease it might be argued that not only the classic infantile cases, but also those that fall ill in (early) childhood may benefit from screening. Neonatal screening for Pompe disease would, however, violate the autonomy of older minors and future adults to decide for themselves whether they would want information on their risk of developing Pompe disease at an unpredictable age. In genetic testing for adults, autonomy of choice is a key principle; people must decide for themselves whether they want to know their genetic make-up or predisposition for disease. Autonomy of choice also holds for screening as an instrument of public health, even though screening is offered in the best interest of the population and a high uptake may be strived for. In case of genetic testing in minors European standards indicate a minor should have reached a certain age to be able to make an informed decision on having a test him-or herself [23]. For instance in case of a disorder that is manifest in a family and that may cause health problems already in the teenage years timely testing is relevant. As for younger ages, testing should only be considered if there is a clear benefit for the child, for instance to prevent imminent health damage or obtain a diagnosis in case of serious health problems.

### Personal preferences

In our interviews professionals differed in their opinion especially regarding detecting the later onset cases, but also on the potential outcome of early treatment in classic infantile cases. It is not self-evident that neonatal screening for Pompe disease would be in the best interest of the child, let alone the future adult, though benefit can certainly not be precluded. The case of Pompe disease questions the format of neonatal screening as an instrument of public health which would be in the best interest of all and for which a high uptake should be achieved. Rather, it could be argued that personal preferences play a major role in weighing pros and cons. The interviewees from our study sometimes referred to their personal (besides their professional) experiences and preferences. It can be argued that in straightforward cases where screening would entail direct benefit for the infant, public-health authorities can more readily decide to offer screening and strive for a high uptake. In more complex cases, such as broad-phenotype disorders, the question arises whether people shouldn’t be able to decide for themselves whether they want to participate in neonatal screening for such a disorder. This may be dependent on their view of what is in the best interest of their child and on whether they think they can cope with or profit from potentially receiving information that their child might fall ill at some point in time.

### Options in a screening package

The possibility to offer options in the neonatal screening package is not new. In recent years technical and policy developments have led to the inclusion of disorders and outcomes in screening packages around the world that do not neatly fit the original intentions of neonatal screening, for instance the disclosure of information on carrier status. In the Netherlands, sickle cell disease (SCD) was added to the neonatal screening programme during a recent expansion in 2007. The screening test for SCD also detected healthy carriers, and the detection of carrier status was regarded as an unsolicited finding. Though disclosing carrier status information would not be of direct benefit for the infant, and could in fact be regarded as a violation of the infant’s right not to know and autonomy of choice, for the parents knowing the carrier status of their infant could have benefits in view of future reproductive options. Parents were therefore given the option to opt out of receiving carrier status information by ticking a box on the heel-prick card. Since screening for cystic fibrosis has been added to the Dutch programme in 2011, parents can choose whether they want to receive their infant’s carrier status information for that disorder, if detected, as well. The format for stating preferences for both disorders was changed at the same time: a box needs to be ticked to indicate whether parents want or do not want to receive carrier status information.

In a recent US article discussing newborn screening for lysosomal storage disorders including Pompe disease it was also argued to give parents an option [24]. However, here the option for screening was suggested to be subsumed under the heading of a research project, where the protocol should be approved by an institutional review board. Though it was perceived screening may have advantages, a lack of evidence on most notably the natural history and treatment were mentioned as arguments to regard screening for Pompe disease as in fact research. The article suggests parents should be informed about the shortcomings at the moment, so they can decide for themselves whether they feel screening would be in the best interest of their child. We would like to add to that suggestion that research is also needed to study the potential psychological burden of disclosure of information and the best ways to support parents and patients-in-waiting.

The possibilities to give parents an option in the Netherlands may be partly different than the one proposed for the US. In the US neonatal screening is routine and mandated in many states, which may explain the choice to subsume screening under the heading of research. In the Dutch context, neonatal screening is not mandatory, though almost 99% of the newborns are screened each year. Since parents can opt in and opt out of receiving information on the carrier status of their child, in principle also other options could be added. However, until now declining screening was only possible for the complete package and not for individual disorders or a subset of disorders. Perhaps Pompe disease could be considered as a separate category, in which case other disorders could be added for which evidence exists about the advantages of screening, yet personal considerations also play a role. When offering such an additional package it would certainly be a challenge to inform parents adequately about the possibilities of the program while safeguarding the uptake of screening for the core diseases for which screening clearly provides a benefit for the infant.

### Policy

In the coming years policy decisions have to be made on whether neonatal screening for Pompe disease should be made available and in what way. We hope the results from the interviews can contribute to making informed and transparent policy decisions. In making the arguments for or against screening for this kind of broad-spectrum phenotype disorders visible, a wider audience can start to reflect on what benefits and harms may be involved. A public discussion will improve people’s understanding and enable exchange of views. Parents can profit from such a discussion and reflect on what may be the benefit or harm in their particular situation, so they may make a better informed decision in case neonatal screening for Pompe would be added to the programme.

## Conclusions

In case of screening for Pompe disease, according to professionals, advantages for both classic infantile and later manifesting, less severe, cases included health gain, avoiding a diagnostic quest, having a reproductive choice in future pregnancies, and the ability to gain more knowledge about the disease and treatment. Being prepared was mentioned as an advantage for the later manifesting cases. Disadvantages for both forms included costs and uncertainties about the effect of treatment. In later manifesting cases most notably the timing of treatment and the psychological burden for the patient-in-waiting and the family were seen as major drawbacks. Facing a reproductive option was sometimes also mentioned as drawback. Requirements for screening included proper information and support for parents, education for health care professionals and screeners. Ideally a test should yield only limited false positive and false negative cases and be able to differentiate between the different forms of Pompe disease. However, for some interviewees the lack of discriminative power was not a reason not to screen.

Professionals draw different conclusions when weighing pros and cons. Some would opt for screening since for them the benefit for classic infantile cases outweighs the potential burden for patients with a late disease manifestation. Others would decline screening because they are of the opposite opinion, or for them there would be too many uncertainties as to the evidence and onset of treatment. Personal preferences and views on the ethical and social ramifications play an important role in considering screening for this broad-phenotype condition, where screening is not necessarily perceived to be in the best interest of all.

## Competing interests

MC is Chair of the Dutch Program Committee Neonatal Heel-prick Screening ( http://www.rivm.nl/pns/hielprik).

Salaries of SW, TR and CE were funded by a grant through Top Institute Pharma, which was financially supported by Genzyme Corporation, the Dutch Health Care Insurance Board (College voor Zorgverzekeringen), Shire Corporation, the Dutch Steering Committee on Orphan Drugs, Erasmus MC University Medical Center, Utrecht University Medical Center, and the Academic Medical Center at the University of Amsterdam. The corporate sponsors of this research played no role in the design of the study, review and interpretation of data, or preparation or approval of the manuscript; they only reviewed the manuscript for intellectual property issues.

SW had a part-time appointment as policy officer at the Dutch Association for Neuromuscular Diseases. As of August 2004, AP and AR provide consulting services for Genzyme Corp, Cambridge, MA, USA, under an agreement between Genzyme Corp and Erasmus MC, Rotterdam, the Netherlands. This agreement also caters to financial support for Erasmus MC for research in Pompe’s disease. Erasmus MC and inventors for the method of treatment of Pompe’s disease by enzyme replacement therapy receive royalty payments pursuant to Erasmus MC policy on inventions, patents and technology transfer.

## Authors’ contributions

MC supervised the project, assisted with designing the interview protocol and reviewed the manuscript. CE designed the interview protocol and code list together with TR, conducted part of the interviews and coded part of the interviews, did the analysis and drafted the text. AP and AR assisted with the development of the interview protocol and the selection of interviewees, and reviewed the manuscript. TR invited the participants, designed the interview protocol and code list together with CE, conducted all interviews and coded part of the interviews, helped with the analysis and the drafting of the text. SW wrote the project proposal and assisted with writing the interview protocol, the selecting of interviewees and the drafting of the text. All authors read and approved the final manuscript.

## Pre-publication history

The pre-publication history for this paper can be accessed here:

http://www.biomedcentral.com/1471-2431/14/203/prepub

## Supplementary Material

Additional file 1 Invitation letter and background information for interviewees. Click here for file

## References

[B1] Burgard P Rupp K Lindner M Haege G Rigter T Weinreich SS Loeber JG Taruscio D Vittozzi L Cornel MC Hoffmann GF Newborn screening programmes in Europe; arguments and efforts regarding harmonization. Part 2 - from screening laboratory results to treatment, follow-up and quality assurance J Inherit Metab Dis 2012 35 613 625 22544437 10.1007/s10545-012-9484-z

[B2] Wilson JMG Jungner G Principles and practice of screening for disease 1968 Geneva: WHO

[B3] Chien YH Chiang SC Zhang XK Keutzer J Lee NC Huang AC Chen CA Wu MH Huang PH Tsai FJ Chen YT Hwu WL Early detection of pompe disease by newborn screening is feasible: results from the Taiwan screening program Pediatrics 2008 122 e39 e45 18519449 10.1542/peds.2007-2222

[B4] Mechtler TP Stary S Metz TF De Jesús VR Greber-Platze S Pollak A Herkner KR Streubel B Kasper DC Neonatal screening for lysosomal storage disorders: feasibility and incidence from a nationwide study in Austria Lancet 2012 379 335 341 22133539 10.1016/S0140-6736(11)61266-X

[B5] Duffey TA Bellamy G Elliott S Fox AC Glass M Turecek F Gelb MH Scott CR A tandem mass spectrometry triplex assay for the detection of fabry, pompe, and mucopolysaccharidosis-I (hurler) Clin Chem 2010 56 1854 1861 20940330 10.1373/clinchem.2010.152009 PMC3442157

[B6] Secretary’s Discretionary Advisory Committee for Heritable Disorders in Newborns and Children (DACHDNC) External evidence review report [ http://www.hrsa.gov/advisorycommittees/mchbadvisory/heritabledisorders/nominatecondition/workgroup.html]. (Accessed December 11, 2013)

[B7] Genetic Alliance Federal advisory committee recommends pompe disease for newborn screening [ http://www.babysfirsttest.org/sites/default/files/Pompe_Final_0.pdf]. (Accessed December 11 2013)

[B8] Cornel MC Rigter T Weinreich SS Burgard P Hoffmann GF Lindner M Loeber JG Rupp K Taruscio D Vittozzi L A framework to start the debate on neonatal screening policies in the EU - an expert opinion document Eur J Hum Genet 2014 22 12 17 23652378 10.1038/ejhg.2013.90 PMC3865414

[B9] Güngör D Reuser AJJ How to describe the clinical spectrum in pompe disease ? Am J Med Genet 2013 Part A 161A 399 400 23300052 10.1002/ajmg.a.35662

[B10] Bombard Y Miller FA Hayeems RZ Avard D Knoppers BM Reconsidering reproductive benefit through newborn screening: a systematic review of guidelines on preconception, prenatal and newborn screening Eur J Hum Genet 2010 18 751 760 20197792 10.1038/ejhg.2010.13 PMC2987364

[B11] Forman J Coyle F Levy-Fisch J Roberts P Terry S Legge M Screening criteria: the need to deal with new developments and ethical issues in newborn metabolic screening J Community Genet 2013 4 59 67 23055099 10.1007/s12687-012-0118-9 PMC3537969

[B12] Potter BK Avard D Wilson BJ Newborn blood spot screening in four countries: stakeholder involvement J Public Health Policy 2008 29 121 142 18368024 10.1057/palgrave.jphp.3200161

[B13] Bruni RA Laupacis A Martin DK for the University of Toronto Priority Setting in Health Care Research Group Public engagement in setting priorities in health care CMAJ 2008 179 15 18 18591516 10.1503/cmaj.071656 PMC2464477

[B14] Weinreich SS Rigter T Van El CG Dondorp WJ Kostense PJ Van der Ploeg AT Reuser AJ Cornel MC Hagemans ML Public support for neonatal screening for pompe disease, a broad-phenotype condition Orphanet J Rare Dis 2012 7 15 22413814 10.1186/1750-1172-7-15 PMC3351372

[B15] Health Council of the Netherlands Neonatal screening 2005 The Hague: Health Council of the Netherlands 11

[B16] Van den Hout HM Hop W Van Diggelen OP Smeitink JA Smit GP Poll-The BT Bakker HD Loonen MC de Klerk JB Reuser AJ Van der Ploeg AT The natural course of infantile Pompe’s disease: 20 original cases compared with 133 cases from the literature Pediatrics 2003 112 332 340 12897283 10.1542/peds.112.2.332

[B17] Rigter T Weinreich SS Van El CG De Vries JM Van Gelder CM Güngör D Reuser AJ Hagemans ML Cornel MC van der Ploeg AT Severely impaired health status at diagnosis of pompe disease: a cross-sectional analysis to explore the potential utility of neonatal screening Molecular Genet Metab 2012 107 448 455 10.1016/j.ymgme.2012.09.017 23040796

[B18] Kwon JM Steiner RD " I’m fine; I’m just waiting for my disease". the new and growing class of presymptomatic patients Neurology 2011 77 522 523 21753177 10.1212/WNL.0b013e318228c15f

[B19] Health Care Insurance Board of the Netherlands Advice alglucosidase Alfa (myozyme) in case of pompe disease(in Dutch) [ http://www.zorginstituutnederland.nl/binaries/content/documents/zinl-www/documenten/publicaties/rapporten-en-standpunten/2012/1211-advies-alglucosidase-alfa-myozyme-bij-de-indicatie-ziekte-van-pompe/1211-advies-alglucosidase-alfa-myozyme-bij-de-indicatie-ziekte-van-pompe/Advies+alglucosidase+alfa+%28Myozyme%29+bij+de+indicatie+%27ziekte+van+Pompe%27.pdf]. (Acccessed August 2014)

[B20] Schippers EI Letter from the minister of health, welfare and sports to the house of representatives of the Netherlands on reimbursement for orphan drugs for pompe disease and fabry diseasedated 30-1-2013, reference number GMT-3152045 (in Dutch) [ http://www.rijksoverheid.nl/documenten-en-publicaties/kamerstukken/2013/01/30/kamerbrief-over-vergoeding-weesgeneesmiddelen-voor-ziekte-van-pompe-en-ziekte-van-fabry.html]. (Acccessed August 2014)

[B21] Van El CG Pieters T Cornel M Genetic screening and democracy: lessons from debating genetic screening criteria in the Netherlands J Community Genet 2012 3 79 89 22109908 10.1007/s12687-011-0063-z PMC3312946

[B22] Van der Ploeg AT Clemens PR Corzo D Escolar DM Florence J Groeneveld GJ Herson S Kishnani PS Laforet P Lake SL Lange DJ Leshner RT Mayhew JE Morgan C Nozaki K Park DJ Pestronk A Rosenbloom B Skrinar A Van Capelle CI Van der Beek NA Wasserstein M Zivkovic SA A randomized study of alglucosidasealfa in late-onset Pompe’s disease N Engl J Med 2010 362 1396 1406 20393176 10.1056/NEJMoa0909859

[B23] Borry P Evers-Kiebooms G Cornel MC Clarke A Dierickx K Genetic testing in asymptomatic minors: background considerations towards ESHG recommendations Eur J Hum Genet 2009 17 711 719 19277061 10.1038/ejhg.2009.25 PMC2947094

[B24] Friedman Ross L Newborn screening for lysosomal storage diseases: an ethical and policy analysis J Inherit Metab Dis 2012 35 627 634 22189599 10.1007/s10545-011-9435-0

