# Supplementary material for: Newborn screening for pompe disease? a qualitative study exploring professional views
Source: BMC Pediatr. 2014 Aug 14;14:203. doi: 10.1186/1471-2431-14-203 (PMC4139142; doi:10.1186/1471-2431-14-203)
Supplement: Additional file 1 — Invitation letter and background information for interviewees. [file 1471-2431-14-203-S1.doc]

**Additional files**

Letter of invitation (sent by e-mail)

Dear…………,

The research group Community Genetics at the VU University Medical Center in Amsterdam, in conjunction with the Pompe Center at Erasmus Medical Center in Rotterdam, recently started research on the desirability of possibly expanding heel prick screening to include Pompe disease. In the context of this research I cordially invite you for an interview.

In the included attachment you can read more about the background of this research.

Because of your knowledge and experience in the area of … I am especially interested in your opinion on this matter and I would very much appreciate your participation in the interview. The purpose of the interview is twofold:

1. to explore views of professionals on this subject
2. to chart the chain of care which would be needed, should this screening be implemented.

The interview will take about one hour and will be recorded in its entirety using a voice-recorder. The information gathered will be processed anonymously and the analysis will eventually lead to publication of the results.

If you wish to co-operate with this research please respond to this e-mail address

([*address not shown here*], the sender of this letter). You may already indicate several possible dates and times when I might schedule the interview. My own preference is [date] or alternatively [date]. Would you be so kind as to provide your telephone number and, if applicable, visiting address? I am happy to travel to you for the interview, but should you be in the vicinity of VU University Medical Center in the near future, we could also hold the interview here.

If you prefer not to participate, I would much appreciate your letting me know via the above e-mail address.

Thanking you in advance,

Yours sincerely,

*[name and contact details of researcher, omitted here]*

Background information provided together with the invitation letter:

**Addendum: research on newborn screening for Pompe disease**

Pompe disease is an autosomal recessive, hereditary disorder caused by a mutation in the alpha-glucosidase gene. The greatly reduced alpha-glucosidase activity in the lysosomes of muscle cells causes glycogen to accumulate, leading to progressive muscle weakness and eventually possible loss of function in cardiac or respiratory muscles. The disease does not manifest at the same age in all patients and the severity differs per patient. In newborns the disease occurs in a very severe form (also called the classic infantile form), with progressive cardiomyopathy and a very short life expectancy (<1 year), and additionally the disease occurs in children and adults in a milder form with moderate to severe muscle weakness and less severe cardiological problems (the more slowly progressive form), sometimes manifesting around the age of sixty.

For more information see www.pompecenter.nl

As you may know, enzyme replacement therapy has been available for patients with Pompe disease since 2006. The treatment results in the first groups of patients have been variable, but mostly promising.

In most patients with the more slowly progressive form, muscle strength stabilizes or improves. In the classic infantile variant of the disease cardiac function in particular seems to improve.

Furthermore an earlier start of treatment seems to lead to a better outcome.

Currently the classic infantile form of Pompe disease is ascertained at a median age of 5 months and more slowly progressive patients are often not diagnosed until years after their first symptoms. Shortening the period between birth and diagnosis therefore seems relevant.

Furthermore, because a promising technique is being developed for distinguishing Pompe patients from non-patients by measuring alpha-glucosidase activity in so-called blood spots, newborn screening may be promising. Currently blood spots are already being collected from all newborns, for screening for 17 other serious treatable diseases.

To obtain a clear idea of the desirability of expanding newborn screening to include Pompe disease, a broad research project was initiated. The goal is to obtain an overview of both the analytical and clinical validity, the clinical utility and the ethical/legal and social aspects. The last two aspects will be investigated among other things by interviews with various medical specialists and professionals.

The research at hand is part of the activity “Improving and developing of methods for early diagnosis of various lysosomal storage disorders” within the project “Sustainable orphan drug development through registries and monitoring” at the Top Institute Pharma ([www.tipharma.com](http://www.tipharma.com/), project no. T6-208), financially supported by the Dutch Health Care Insurance Board, the [Dutch] Steering Committee on Orphan Drugs, Genzyme Corporation, Shire Corporation, the Academic Medical Center at the University of Amsterdam, Erasmus MC University Medical Center (Rotterdam) and Utrecht University Medical Center.

In principle the Health Council of the Netherlands uses as a criterion for heel prick screening that it must provide direct benefit for the newborn child. For patients with the classic infantile form of Pompe disease the benefit of newborn screening seems self-evident, but heel prick screening for Pompe disease also entails detection of more slowly progressive variants. The question is, what does this means for these currently asymptomatic patients and for their care? Do the benefits for both forms of Pompe disease outweigh the possible disadvantages?
